# Supplementary material for: The interactome of intact mitochondria by cross-linking mass spectrometry provides evidence for coexisting respiratory supercomplexes
Source: Mol Cell Proteomics. 2017 Dec 8;17(2):216–32. doi: 10.1074/mcp.RA117.000470 (PMC5795388; doi:10.1074/mcp.RA117.000470)
Supplement: Supplemental Data [file supp_RA117.000470_133922_0_supp_23978_fzffwf.zip › spectra_annotation/mito_spectra_annotation/107-1-7-1-1-1.pdf]

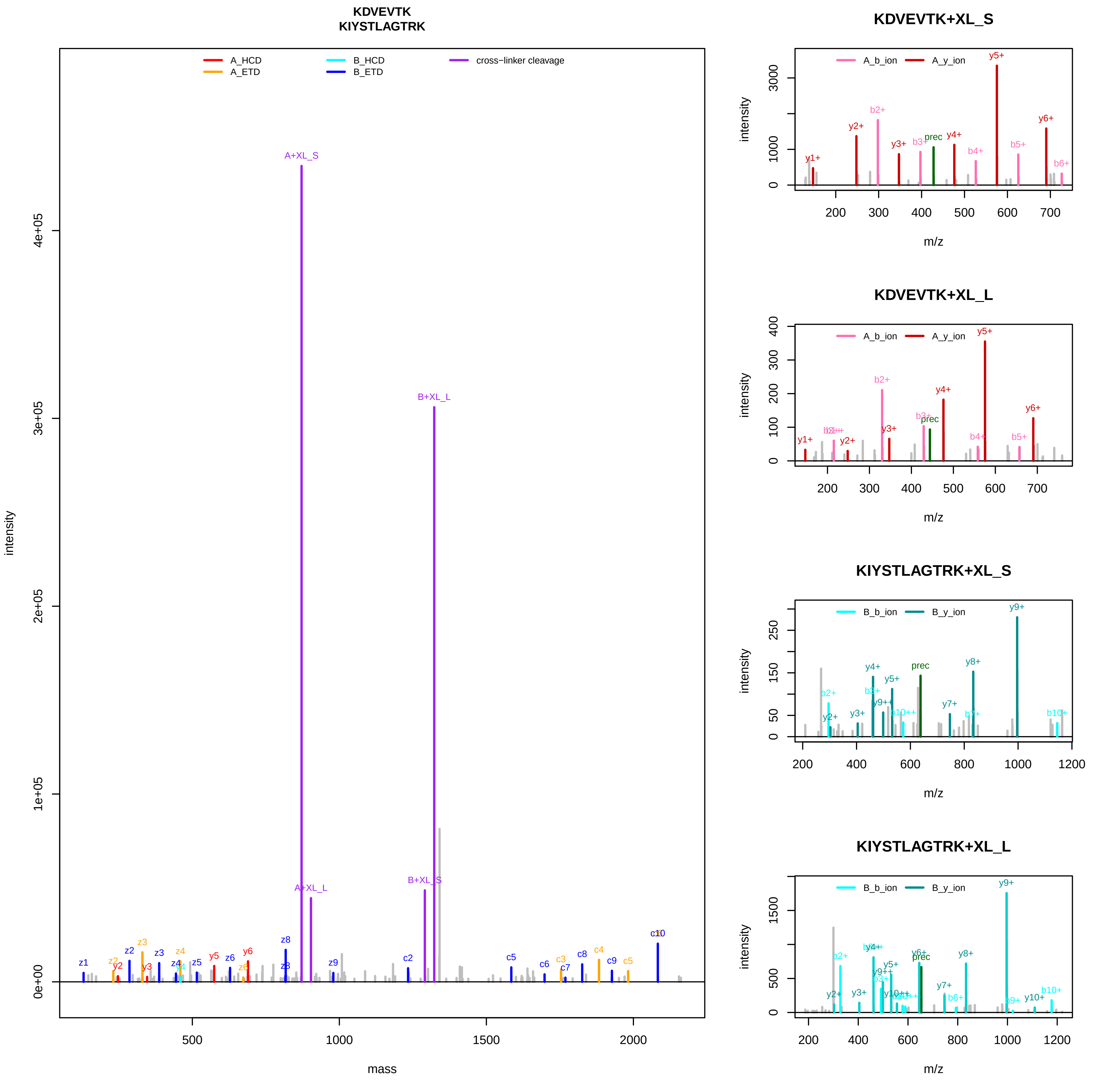
KDVEVTK
KIYSTLAGTRK
A_HCD
A_ETD
B_HCD
B_ETD
cross−linker cleavage
intensity
mass
0e+00
1e+05
2e+05
3e+05
4e+05
500
1000
1500
2000
A+XL_S
B+XL_L
A+XL_L
B+XL_S
z1
z2
y2
z3
y3
z4
z5
y5
z6
y6
z8
z9
c2
c5
c6
c3
c7
c8
c4
c9
c5
c10
KDVEVTK+XL_S
KDVEVTK+XL_L
KIYSTLAGTRK+XL_S
KIYSTLAGTRK+XL_L
A_b_ion
A_y_ion
B_b_ion
B_y_ion
prec
m/z
y1+
y2+
b2+
y3+
b3+
y4+
b4+
y5+
b5+
y6+
b6+
y7+
y8+
y9+
b10+
